# Trypanosomosis, Its Risk Factors, and Anaemia in Cattle Population of Dale Wabera District of Kellem Wollega Zone, Western Ethiopia

**DOI:** 10.1155/2014/374191

**Published:** 2014-09-16

**Authors:** Habtamu Biyazen, Reta Duguma, Mebratu Asaye

**Affiliations:** ^1^College of Agro-Industry and Land Resource, Haramaya University, P.O. Box 226, Chiro, Ethiopia; ^2^College of Veterinary Medicine and Agriculture, Addis Ababa University, P.O. Box 34, Debre Zeyit, Ethiopia

## Abstract

Cross-sectional study was conducted in Dale Wabera district of Kellem Wollega zone, Western Ethiopia, to determine the prevalence of bovine trypanosomosis and to evaluate association of bovine trypanosomosis to anaemia. Blood samples collected from 384 randomly selected cattle were subjected to parasitological and haematological analysis. For the parasitological survey, blood samples were examined using a buffy coat technique. The packed cell volume (PCV) value of each animal was also measured using hematocrit reader. The overall prevalence of trypanosomosis was 2.86%. The most common trypanosome species identified were *Trypanosoma congolense* (63.64%) followed by *T. vivax* (27.27%) and *T. brucei* (9%). The prevalence showed no significant difference in susceptibility between sex categories, age groups, and different body conditioned animals. The overall anaemia prevalence in the area was 19.27%. The anaemia prevalence was significantly higher in trypanosome positive cattle (54.54%) than in noninfected animals (18.23%) (*P* < 0.05). The mean PCV value of the infected animals was lower (22.36% ± 7.39%) compared to noninfected animals (27.86 ± 5.38%). There was statistically significant difference (*P* < 0.05) in the PCV values of infected and noninfected animals. In conclusion, this study confirmed that trypanosomosis poses a threat to cattle production in the area and also contributed to the occurrence of anaemia.

## 1. Introduction

Trypanosomosis is a widely spread protozoan disease complex which affects cattle and other wide range of hosts in sub-Saharan Africa. The course of the disease may run from a chronic long lasting to an acute and rapidly fatal depending on the vector-parasite-host interactions. The disease is mainly characterized by intermittent fever, progressive anaemia, and loss of condition of susceptible hosts which if untreated leads to heavy mortalities [1]. The etiological agent of the disease is unicellular flagellated protozoan parasite of a genus* Trypanosoma*. Trypanosomes are blood borne unicellular protozoan parasites dwelling in various body and tissue fluids. The parasite is known for more than a century, but still control of the disease remains elusive [2].

Several species of hematophagous tsetse flies of the genus* Glossina* are the vectors of African trypanosomosis and are responsible for cyclical transmission of the parasitic protozoan between numerous vertebrate hosts. The vector is distributed over wide range of habitats covering about 10 million square kilometers of potential grazing lands in 37 countries which are rendered unsuitable for livestock breeding and farming across the African content [3], exposing 160 million cattle to the risk of anaemia, emaciation, and death and 55 million people to the risk of fatal sleep [4]. As a result agriculture revolution which is a key element in the fight against poverty and the improvement of food security in developing countries failed in tsetse infested areas of sub-Saharan Africa [5].

In Ethiopia, trypanosomosis is one of the most important diseases that limit livestock productivity and agricultural development due to its high prevalence in the most arable and fertile land of southwest and northwest part of the country following the greater river basins of Abay, Omo, Ghibe, and Baro [6]. Currently about 220,000 km^2^ areas of the above-mentioned regions are infested with five species of tsetse flies, namely,* Glossina pallidipes, G. morsitans, G. fuscipes, G. tachinoides,* and* G. longipennis* [7]. More than 10 million heads of cattle in Ethiopia are at risk of variable degrees of trypanosomosis at any time of the year, of which six million are tsetse borne [8]. A number of studies have been so far undertaken in different parts of the country to determine the magnitude of this economically important disease [9–12]. Nevertheless, there are no published studies which assess the prevalence of this disease in Dale Wabera district.

The distribution of trypanosomes is dynamic due to climatic change, ecological disturbances, and human interventions. Some tsetse infested areas are scarce in infrastructure and devoid of human settlements. In such areas sufficient information is lacking about the status of trypanosomosis. Even in already studied areas updating the prevalence and status of the disease have paramount importance for understanding the epidemiology of the disease, to facilitate the choice of suitable control methods, and to help in planning for development programs in the area. Therefore, the objectives of this study were to determine the prevalence of bovine trypanosomosis in Dale Wabera district and to evaluate association of bovine trypanosomosis with anaemia.

## 2. Materials and Methods

### 2.1. Study Area

The study was conducted in Dale Wabera district of Kellem Wollega zone of Oromia regional state, Western Ethiopia, during late rainy season. The altitude of the area ranges from 1100 to 1800 m.a.s.l. The mean minimum and maximum temperature are 11.0–15.5°C and 26.1–34°C, respectively. The agroclimate of the area alternates between long summer rain fall (June to September) and winter dry season (December to March) with annual rainfall ranging from 1300 to 1600 mm. The livelihood of the society largely depends on mixed livestock and crop production. The total land cover of the district is about 1132.02 km^2^ of which 424.77 km^2^ is infested by tsetse flies [7].

### 2.2. Study Animals

The animals used for this study were local zebu cattle (*Bos indicus*), which are usually kept under an extensive husbandry system. The studied cattle were herded together during the day time and returned to their individual owner's farmstead each evening. Their age was categorized into three age groups (<2 years, 2–5 years, and greater than five years) based on dentition and the body condition score was grouped into poor, medium, and good conditioned animals based on the appearance of ribs and dorsal spines applied for zebu cattle [13].

### 2.3. Sample Size Determination

The animals were sampled randomly involving both sexes, all age groups, and all types of body conditions. The desired sampling size was calculated according to the formula given by [14]. As there have been no published studies reported in this area, the sample size was determined based on the expected prevalence of 50%, confidence level of 95%, and 5% desired absolute precision. As result a total of 384 cattle were sampled from ten different locations (villages) in the district.

### 2.4. Study Methodology

#### 2.4.1. Packed Cell Volume (PCV) Determination

Blood samples were obtained by puncturing the marginal ear vein with a lancet and collected directly into a pair of heparinised capillary tubes. After centrifugation at 12,000 rpm for 5 min in a microhaematocrit centrifuge, the capillary tubes were placed in a haematocrit reader and the length of the red cells column was expressed as a percentage of the total volume of blood. Animals with PCV less than 24% were considered to be anaemic [4].

#### 2.4.2. Buffy Coat Technique (BCT)

Heparinised capillary tubes, containing blood samples, were cut using a diamond tipped pen 1 mm below and 3 mm above the buffy coat after centrifugation. The content of the capillary tube was expressed onto a glass slide, then covered with cover slip, and examined under ×40 objective and ×10 eye piece for movement of parasite. Trypanosome species were identified according to their movement in wet film preparations as provided by [4].

### 2.5. Data Analysis

Prevalence was analyzed by determining total positive cases out of the total number of animals sampled. Infection rate on the basis of sex, age, and body condition was compared using *χ* ^2^ test (chi-square). Mean PCV in parasitemic and aparasitemic animals was compared using *t*-test. Significance test was set at 5% alpha and 95 confidence interval.

## 3. Results

### 3.1. Parasitological Findings

**Figure 1:**
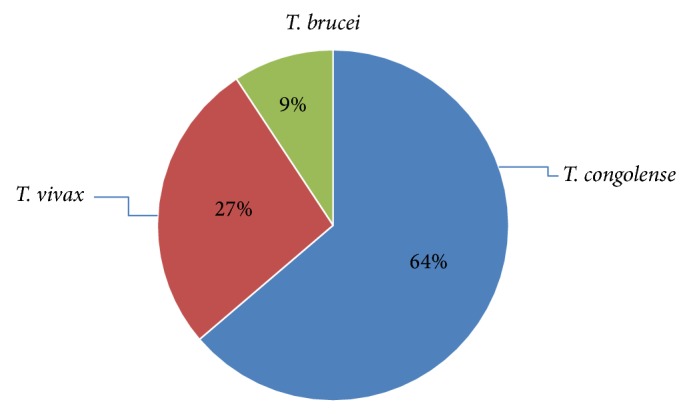
Distribution of the species of trypanosomes among the infected animals.

Out of 384 cattle examined 11 (2.86%) were found to be infected with trypanosomes. The prevalence in terms of trypanosome species was 1.82%* T. congolense*, 0.78%* T. vivax*, and 0.26%* T. brucei.* The proportion of trypanosome species was 63.64% (7/11)* T. congolense*, 27.27% (3/11)* T. vivax,* and 9% (1/11)* T. brucei* (Figure 1). During study period mixed infection was not detected.

*Prevalence of Trypanosomosis according to Age, Sex, and Body Condition*. The prevalence of trypanosomosis was higher in males (3.64%) as compared to female animals (1.45%) (Table 1). However, the difference was not statistically significant (*P* > 0.05). The highest prevalence was observed in the adult animals greater than 5 years old (Table 1) and the variation in prevalence between the different age groups was also not statistically significant (*P* > 0.05). The prevalence of trypanosomosis between body condition scores was 3.37% in poor, 2.81% in medium, and 2.61% in good body conditioned animals and it was statistically not significant (*P* > 0.05) as indicated in Table 1.

### 3.2. Cattle PCV Distribution in Studied Area

**Figure 2:**
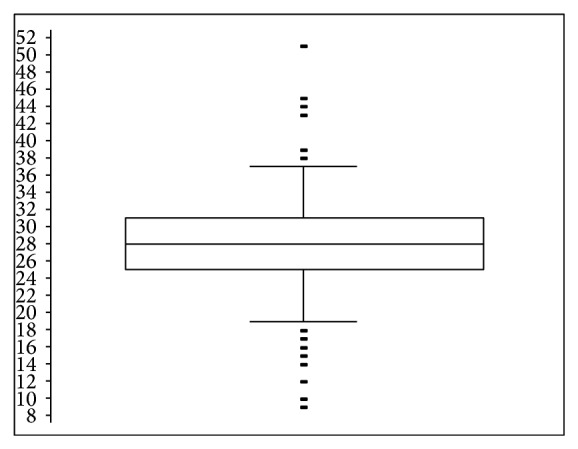
PCV distribution in cattle population of Dale Wabera district.

The frequency distribution of PCV % for the overall studied 384 cattle is indicated in Figure 2. The mean PCV value of 27.7% was registered during the study period. The most frequently recorded PCV value was 28% and was recorded in 35 cattle from the overall studied animals in the district. The mean PCV values of cattle were significantly (*P* = 0.0011) influenced by trypanosome infection as 27.86 and 22.36% PCV values in trypanosome positive and trypanosome negative animals were registered, respectively (Table 2).

### 3.3. Prevalence of Trypanosomosis and Its Share in Prevalence of Cattle Anaemia

The overall anemia prevalence in the studied district was 19.27% (74/384). The anaemia prevalence was significantly higher in trypanosome positive cattle (54.54%) than in noninfected cattle (18.23%) (*P* < 0.05). Of 19.27% anaemia prevalence, 1.56% (6/384) was trypanosome infected animals. However, large number of animals 17.7% (68/384) had anaemia (PCV < 24) without having trypanosome infection. Some animals 1.3% (5/384) were infected by trypanosome but their PCV was found normal (Table 3).

## 4. Discussion

The overall prevalence of bovine trypanosomosis in the study area was 2.86%. The finding of the current study is lower than a range of studies conducted previously in Ethiopia: Tafese et al. [15] studied prevalence of bovine trypanosomosis in East Wollega zone using buffy coat technique and found prevalence rate of 8.5%; Mekuria and Gadissa [16] reported 12.41% prevalence in Metekel and Awi zones of Northwest Ethiopia. Cherenet et al. [9], who assessed cattle trypanosomiasis in the tsetse-free and the tsetse-infested zones of the Amhara region of Northwestern Ethiopia using molecular diagnostic method, reported infection rates of 20.9% and 25.7%, respectively. This result was also lower as compared to [6] at tsetse infested areas of Ethiopia (17.67%); [17] in Dembecha and Jabitehenan (12%); and [18] in Metekel district (17.20%).

Lower prevalence was found in this study compared to the works of these authors elsewhere in the country. This disparity emanates from many factors that explain the lower trypanosomosis prevalence in the study area. There were parasite and vector control programmes practiced in the area. Also as the study was conducted during late rainy season it is obvious that the population of flies increases. Due to this farmers inject their animals with trypanocidal drugs and also use insecticide spray in this season better than any other time to minimize the effect of the disease. These results in the lower prevalence of trypanosomosis observed in this study. In addition, expansion of veterinary services up to peasant association and deforestation for crop cultivation and settlement might also have contributed to the low prevalence. The lower prevalence observed in this study could also be due to inadequacy of parasite detection method used. It was reported that the buffy coat microscopy technique is relatively an insensitive diagnostic method as it fails to detect 66% of infected cattle [19]. The molecular diagnostic techniques which permit precise identification of the parasite to species level and serological diagnostic methods are more sensitive [20].

Out of the 2.86% overall prevalence of trypanosome infection, 1.82% were due to* T. congolense*, 0.78% were due to* T. vivax*, and 0.26% were due to* T. brucei.* The finding of this study showed that of the total trypanosome positive animals 63.6% were found to be infected with* T. congolense*, 27.2% were infected with* T. vivax*, and the remaining 9% were infected with* T. brucei.* In the current study mixed infection was not detected. The higher proportion of* T. congolense* in this study was in agreement with the previous results of [6] for tsetse infested areas of Ethiopia (58.5%) and [21] at Mereb Abaya, South Ethiopia (66.1%). Moreover, the results of [22] at Arba Minch Zuria districts (85.2%) and [23] in Ghibe valley, Southwest Ethiopia (84%), had also shown higher results of* T. congolense*.

The predominance of* T. congolense* infection in cattle suggests that the major cyclical vectors or* Glossina* species are more efficient transmitters of* T. congolense* than* T. vivax* in East Africa [24] and also due to the high number of serodems of* T. congolense* as compared to* T. vivax* and the development of better immune response to* T. vivax* by infected animals [25]. Different studies [25, 26] have indicated that* T. vivax* is highly susceptible to treatment while the problems of drug resistance are higher in* T. congolense*, and* T. congolense* is mainly confirmed in the blood, while* T. vivax* and* T. brucei* also invade the tissues [27]. According to [6],* T. congolense* and* T. vivax* are the most prevalent trypanosomes that infect cattle in tsetse infested and tsetse free areas of the Ethiopia, respectively.

The prevalence of bovine trypanosomosis was studied in different sex, body condition, and age groups of cattle and significant variation was not observed (*P* > 0.05). This might be because of an equal chance of exposure to the parasite. This result is in agreement with the previous researches reported by [10, 12, 15, 28]. In the present study sex was not found to be the risk factor. This finding could be during late rainy season as plough stress for males reduced; both males and females can be affected uniformly in high tsetse challenge areas.

The overall anaemia prevalence in the studied district was 19.27%. When infected and noninfected animals were compared, the anaemia prevalence was significantly higher in trypanosome positive cattle (6/11, 54.54%) than in noninfected cattle (68/373, 18.23%) (*P* < 0.05). This finding was in agreement with previous reports [9, 10, 28]. Of total anaemia prevalence (19.27%), 1.56% was trypanosome positive animals. However, large number of animals, 17.7%, had anaemia without having trypanosomosis infection. This suggests that even though anaemia is characteristic of trypanosomosis, other factors are also anticipated to affect the PCV profile of animals. Diseases such as fasciolosis, gastrointestinal parasitism, vector-borne diseases, and nutritional deficiencies can also cause reduced PCV [29]; however there were no previous published research reports of these diseases in the studied area.

Some animals were infected by trypanosome but their PCV was normal and anaemia was not recorded in them. This might be due to some infected animals being able to keep their PCV within the normal range for a certain period of time. The appearance of parasitologically negative animals with PCV values of less than the threshold value set (24%) may be due to inadequacy of the detection method used [20], other anaemia causing diseases [29], or delayed recovery of the anaemic situation after current treatment with trypanocidal drugs. Furthermore, the occurrence of positive animals with PCV of greater than 24% might be thought of as recent infections of the animals [29].

The mean PCV value of parasitemic animals was found to be significantly lower (22.36% ± 7.39) than that of aparasitemic (27.86% ± 5.38) animals which is similar to the results obtained by [9, 28, 30]. Taking the PCV value 24 to 46% as normal for zebu cattle [31], 54.5% of the parasitemic and 18.2% aparasitemic animals have registered PCV values less than 24%. Low PCV value may not solely be due to trypanosomosis. However, these factors are likely risks for both parasitaemic and nonparasitaemic animals. Therefore the difference in mean PCV value between parasitemic and aparasitemic animals indicates that trypanosomosis is involved in reducing the PCV values in the infected animals.

## 5. Conclusion

This study indicated that trypanosomosis is an important disease and a potential threat that affects the health and productivity of cattle in Dale Wabera district. The major species of trypanosomes in the study area were* T. congolense* followed by* T. vivax* and* T. brucei*. Nearly 20% of the sampled animals had a PCV value of below 24% and were thus considered as anaemic. The anaemia prevalence was significantly higher in trypanosome positive cattle than in noninfected cattle. The mean PCV value of parasitemic animals was significantly lower (22.36% ± 7.39) than that of aparasitemic (27.86% ± 5.38) animals. This indicates that infection with trypanosomosis negatively affects PCV profile of animals. Therefore, proper strategies have to be designed and implemented to minimize its effect on livestock production in the studied area.

## Figures and Tables

**Table 1:** Prevalence of bovine trypanosomosis according to sex, age, and body condition score in Dale Wabera district.

Host related risk factors	Number of examined cattle	Number of infected cattle	Prevalence (%)	*χ* ^2^	*P* value
Sex					
Female	13	2	1.45	1.485	0.223
Male	247	9	3.64		
Total	**384 **	**11 **	**2.86 **		
Age					
<2 year	25	0	0	3.397	0.183
2–5 years	158	4	2.53		
>5 years	201	7	3.48		
Total	**384 **	**11 **	**2.86 **		
Body condition					
Good	153	4	2.61	0.112	0.945
Medium	142	4	2.81		
Poor	89	3	3.37		
Total	**384 **	**11 **	**2.86 **		

**Table 2:** Mean PCV comparison between infected and noninfected animals.

Condition	Number	Mean	SD	*t*-test	*P* value
Infected	11	22.36	7.3929	3.3020	0.0011
Noninfected	373	27.86	5.3814		

**Table 3:** Proportion of anaemia from trypanosome infected and noninfected cattle population.

Trypanosome	Anemia	Frequency	Percent	Percent share per strata
Noninfected	Negative	305	79.4%	81.8%
	Positive	68	17.7%	18.2%

Infected	Negative	5	1.3%	45.5%
	Positive	6	1.5%	54.5%
